# Rapid Processing of Wafer-Scale Anti-Reflecting 3D Hierarchical Structures on Silicon and Its Templation

**DOI:** 10.3390/ma11122586

**Published:** 2018-12-18

**Authors:** Harsimran Singh Bindra, Jaikrishna R., Tushar Kumeria, Ranu Nayak

**Affiliations:** 1 Amity Institute of Nanotechnology, Amity University Uttar Pradesh, Noida 201301, UP, India; harsimransinghbindra@gmail.com (H.S.B.); rjai.1175@gmail.com (J.R.); 2 School of Pharmacy, The University of Queensland, Brisbane, QLD 4102, Australia

**Keywords:** silicon, hierarchical structure, anti-reflection, metal assisted chemical etching, electroless deposition

## Abstract

Hierarchically structured silicon (Si) surfaces with a combination of micro/nano-structures are highly explored for their unique surface and optical properties. In this context, we propose a rapid and facile electroless method to realize hierarchical structures on an entire Si wafer of 3″ diameter. The overall process takes only 65 s to complete, unlike any conventional wet chemical approach that often combines a wet anisotropic etching of (100) Si followed by a metal nanoparticle catalyst etching. Hierarchical surface texturing on Si demonstrates a broadband highly reduced reflectance with average R% ~ 2.7% within 300–1400 nm wavelength. The as-fabricated hierarchical structured Si was also templated on a thin transparent layer of Polydimethylsiloxane (PDMS) that further demonstrated prospects for improved solar encapsulation with high optical clarity and low reflectance (90% and 2.8%).

## 1. Introduction

There has been an exponential growth in the field of micro/nano-texturing of a silicon (Si) surface due to its attractive properties like controllable surface wettability and optical properties [1,2,3]. Especially hierarchical structures with a unique combination of micro/nano-structures on Si that makes them highly useful for applications like self-cleaning, solar cell, photoluminescence, contamination prevention, etc. [3,4,5]. Various techniques like vapor-liquid-solid (VLS), reactive ion etching (RIE), electrochemical etching, and wet anisotropic electroless etching have been used for fabricating micro and nano-structures on Si surfaces [5,6,7,8,9]. However, methods like VLS and RIE are expensive and complicated. Despite the ease and simple process of wet electrochemical and electroless etching, they result in a non-uniform and poorly controllable porous structure. Furthermore, the type of micro/nano pattern generated using wet chemical etching is dependent on the crystal orientation. For example, chemical etching of (100) Si crystal orientation generates pyramid like structures, whereas anisotropic etching of (110) Si results in the generation of micro-structures with vertical side walls [10,11]. The reason behind the growth of pyramid shaped micro-structure in (100) Si is due to the presence of two dangling bonds and two back bonds in each atom of (100) Si. Thus a low activation energy is required to remove an atom from the (100) lattice; hence, (100) Si experiences faster etching with alkaline etchant solution than other surface orientations [12]. Nanotexturing on Si surface using metal nanoparticle assisted etching has also been reported [4,13,14,15,16]. Electroless etching using metal nanoparticles is a rather rapid and facile process. There are several reports where wet chemical anisotropic etching (in potassium hydroxide (KOH)) and metal nanoparticle assisted etching process has been combined to realize hierarchical structures on Si [2,3,17]. Superhydrophobicity with a water contact angle (WCA) as high as 166° on the Si surface using conventional KOH based etching combined with gold nanoparticle assisted etching followed by a treatment with perfluorooctyl trichlorosilane (PFTOS), has been demonstrated in the literature [2]. However, the superhydrophobicity is imparted by the self-assembled monolayer (SAM) of PFTOS that are known to be adversely affected by environmental conditions. Besides superhydrophobicity, hierarchical texturing on Si surface have also been demonstrated to exhibit improved broadband anti-reflection property. The literature has demonstrated a WCA of 165° and an average optical reflectance of 4% over a wavelength of 450–2500 nm on a hierarchically textured Si surface [3]. Other than the metal nanoparticle assisted etching technique, hierarchical structuring on Si has also been described using e-beam lithography inductively coupled with plasma etching, etc. Si surfaces with reflection as low as 2% have been created using these techniques, however, most of these processes are complex, expensive, and time consuming [18,19]. 

Additionally, inverted micro/nano-pyramid structures developed using micro/nano structured Si templates have also shown bright prospects towards light trapping [20,21,22,23,24,25,26,27]. Due to their V-shaped architecture, the incident light experiences multiple internal reflections along the side walls that overall leads to minimized reflection of light than the vertically grown pyramid micro-structures. According to previous literature, micro-scaled inverted pyramidal structures were fabricated by metal nanoparticle assisted etching technique [20,21,22,23], whereas fabrication of nano-scale pyramids employed a combination of wet alkaline etching and photolithography technique [24,25,26,27]. However, due to the complexity and high cost, the photolithography process may not be preferred for high throughput commercial fabrication. The formation of inverted pyramidal structures on Si depends on substrate orientation. This implies the occurrence of faster Si etching initiating along direction (100) and terminating at (111) plane, and overall it forms an inverted pyramidal architecture (upright if etched with an alkaline solution). Comparative to (100) configuration metal assisted etching performed on (110) Si and (111) Si, results in the formation of rhombus shaped and triangular/hexagonal shaped structures, respectively [23]. According to Chen et al. these rhombus and triangular shaped structures formed on (110) Si (110) and (111) Si offers poor light management comparative to inverted pyramid structures formed on (100) Si [23]. 

It is clear that most reports on the anti-reflecting surface of Si involve either a three-step process [3,17,28] or a single step etching of longer duration [21]. Lack of a simple and rapid process to generate anti-reflecting Si surface has motivated us to develop a fast and facile electroless method to fabricate uniform hierarchical micro/nano-structures on Si over a large area. Unlike the previous literature based on the fabrication of hierarchical structure on Si wafer using three-step texturing process; our proposed two-step method was made possible by selecting the unpolished side of a commercial (100) Si wafer that was pre-textured with micron dimension square shaped indents. These square micro-structures often result during the polishing step of a Si wafer during the lapping process. Further, nanotexturing on this micro-structured Si surface using a conventional silver (Ag) nanoparticle assisted etching process resulted in the formation of nanoscale pits on its surface. The as-obtained hierarchical structured Si was further used as a template to pattern a thin PDMS layer for potential application as a solar encapsulation material. Here, we demonstrate the fabrication of a wafer-scale hierarchically textured Si template (3″ diameter) and imprinting of a thin PDMS layer thereof. The patterned PDMS layer was identified to have excellent anti-reflection and high transmittance. 

## 2. Materials and Methods

### 2.1. Materials

A 3″ diameter single side polished (100) plane *n*-type (phosphorus doped) Si wafer (Vinkarola), Sylgard 184 PDMS kit (Dow Corning), hydrofluoric acid (40 wt%), nitric acid (68 wt%), silver nitrate, n-hexane, and hydrogen peroxide (30 wt%) were purchased from Merck (Burlington, MA, USA). For coating, the structurally engineered Si wafer, 1-trichloro (1H,1H,2H,2H-perfluorooctyl) silane, was obtained from Sigma Aldrich (India). All chemicals and salts used were analytical grade. DI water (18 MΩ cm) was used to prepare all the solutions.

### 2.2. Methodology

#### 2.2.1. Fabrication of Hierarchically Structured Si

Si wafers were cleaned using standard RCA cleaning protocol and stored in a vacuum desiccator till further experiments were conducted. The detailed method for metal nanoparticle assisted etching using Ag nanoparticles has been adopted from previous literature [3,14,15,29]. In brief, a mixed aqueous solution of 0.01 M of silver nitrate (AgNO_3_) and 4.6 M hydrofluoric acid (HF) was prepared at room temperature. Important Note: Hydrofluoric acid (HF) is a highly corrosive and toxic acid, thus any exposure to HF should be avoided. The deposition of Ag nanoparticles was performed in dark condition for variable time intervals of 30 s, 60 s, and 120 s. The wafers were gently cleaned and dried before the next processing step. Further, the Si wafers deposited with Ag nanoparticles were etched in a solution of hydrofluoric acid (HF), hydrogen peroxide (H_2_O_2_), and DI water mixed in a volume ratio of 1:5:10 at room temperature. Etching was performed for three different time durations for 5 s, 10 s, and 20 s under dark condition. The etched samples were then washed thoroughly in DI water and further dipped in nitric acid (HNO_3_) to remove the Ag nanoparticles deposited on the Si surface. After repeated washing in DI water and ultrasonication, the etched Si samples were dried and systematically analyzed. The micro/nano-dimension features on the Si surface (SEM image) were measured using ImageJ (free image analysis tool developed by the National Institutes of Health, NIH, USA) software. 

#### 2.2.2. Patterning PDMS

**Scheme 1:**
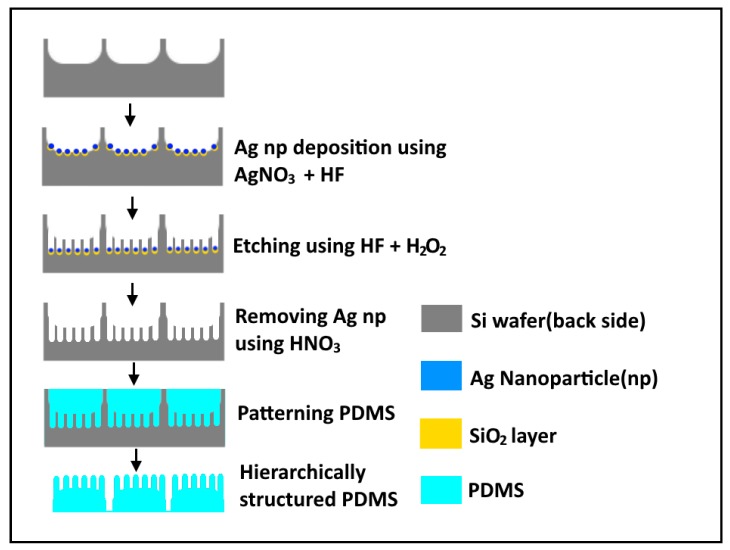
Fabrication technique for texturing the lapped unpolished side of a (100) Si wafer and surface patterning of polydimethylsiloxane (PDMS) using the textured Si template. The process involves deposition of a uniform layer of Ag nanoparticles through an electroless deposition using an aqueous electrolyte mixture of AgNO_3_ and HF followed by treatment with an aqueous solution of HF and H_2_O_2_ for 5 s. This resulted in the generation of a hierarchical micro/nano-structure with excellent broadband antireflection property. The textured Si surface can also be used as a template to prepare light trapping structures in PDMS for photovoltaic applications.

Before spin coating the PDMS pre-polymer mix, the etched Si wafer was treated with a self-assembled monolayer (SAM) of 1-Trichloro (1H,1H,2H,2H-perfluorooctyl) silane to make the Si surface super-hydrophobic so that cured PDMS could be easily peeled off. After pre-treatment of the Si template, PDMS pre-polymer and curing agent were mixed in a ratio of 10:1 *w*/*w* and degassed to remove the bubbles generated during the mixing process. The mixture was then diluted with n-hexane with 1:1 *v*/*v* and spin coated onto the Si wafer and cured at 120 °C for 10 min. The cured PDMS layer was then peeled off from the Si surface. Dilution was necessary to lower the viscosity of the PDMS pre-polymer mix so as to ensure its capillary filling inside the pores of the etched Si. The process of hierarchical patterning of PDMS is schematically illustrated in Scheme 1.

#### 2.2.3. Physical and Optical Characterization

A Zeiss-EVO 18-448Scanning electron microscope. with EDX detector attachment (Zeiss, Germany) was used for structural analysis. UV-Vis-NIR spectrophotometer (Shimadzu-UV 2600, Japan). equipped with integrating sphere (ISR-2600 plus) attachment was used for the transmission and reflection measurements. Optical measurements were measured from five different positions of the etched Si wafer and patterned PDMS. The integrating sphere is equipped with two detectors—photomultiplier tube (PMT) and InGaAs and can scan wavelengths from 220 nm to 1400 nm. During diffused reflectance measurement, the incident beam of light is directed to the sample surface at an incidence angle of 0° and for specular reflectance measurement, the light is directed at an incident angle of 8° on the sample surface. All the transmission measurements performed on the PDMS membrane were a direct measurement. Further, reflection and transmission measurements were acquired at five different positions of the etched Si wafer and patterned PDMS. 

## 3. Results

### 3.1. Structural Analysis of Ag Nanoparticle Deposited Si Wafer

**Figure 1:**
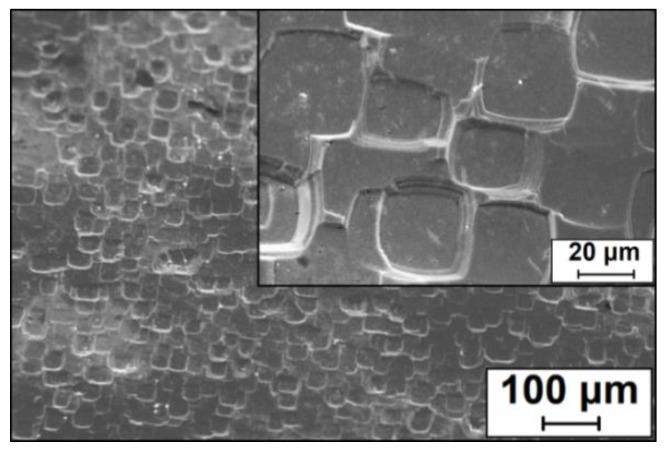
SEM image of the unpolished side of a Si wafer showing regularly distributed square indents; inset shows a higher magnification SEM image.

The scanning electron microscope (SEM) image in Figure 1 shows the surface morphology of the unpolished side of a Si wafer. The unpolished side of the Si wafer comprises of square-shaped indented micro-structure with an average dimension of 25 µm × 35 µm × 3 µm (l × b × h). A magnified view of the lapped micro-structures is shown in the inset. 

**Figure 2:**
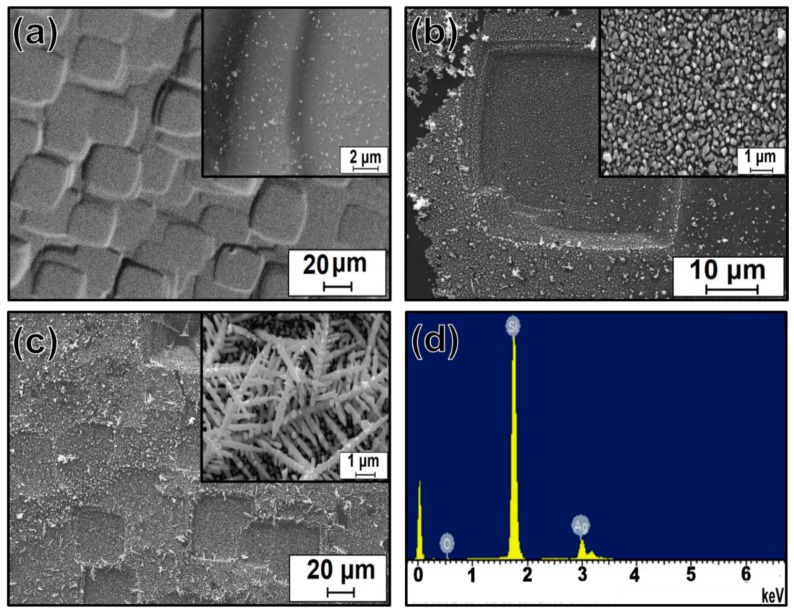
SEM images of Ag nanoparticles deposited on the unpolished side of the Si wafer for time periods (**a**) 30 s; (**b**) 60 s; (**c**) 120 s (Insets: higher magnification SEM); (**d**) EDX analysis of Ag nanoparticle deposited (60 s) Si wafer.

Three different time intervals of 30 s, 60 s, and 120 s were used for electroless deposition of Ag nanoparticles on the unpolished side of the Si wafer. Ag nanoparticles, when deposited for a time span of 30 s, resulted in low surface area coverage on Si as shown in Figure 2a and clearly visible in the magnified view shown in the corresponding inset. Increasing the deposition time to 60 s resulted in an improved surface coverage with uniform inter-particle spacing (Figure 2b). The average dimension of the Ag nanoparticles was measured between 150 nm and 170 nm (using ImageJ) as can be observed from the inset of Figure 2b. On further increasing the time duration up to 120 s, an excess growth of Ag nano-structures was observed as shown in Figure 2c. By doubling the deposition time a vigorous increase in particle growth rate is experienced, however, a more intense particle growth is observed across the edges of square micro-structures. Comparative to inner space, the Ag nanoparticles across the edges were experiencing a faster growth rate and an increased deposition time allowed them to transform into branched structures (inset of Figure 2c). Presence of dendritic Ag micro/nano-structures masked the entire substrate (Si) that limited its exposure during HF etching. Moreover, due to excessive multilayered growth, the Ag dendrites appeared to physically detach from the underlying Si surface. Thus, Ag deposition time of 60 s was chosen due to uniform Ag coverage, controlled moderate interparticle-spacing, and strong adhesion between the nanoparticles and the Si surface. EDX was performed to confirm the formation of Ag nanoparticles on the Si surface. The EDX spectrum shown in Figure 2d clearly indicates deposition of Ag nanoparticles on Si.

**Figure 3:**
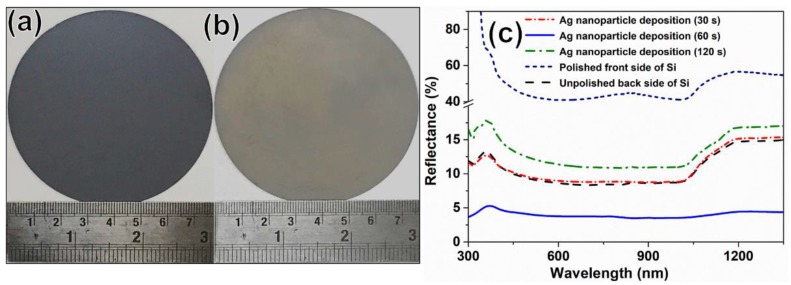
Digital photographs of (**a**) untreated unpolished side of Si wafer; (**b**) Ag nanoparticles deposited (60 s) on the unpolished side of Si wafer showing a significant change in color; (**c**) reflectance spectrum of the unpolished side of Si wafer with Ag nanoparticle deposition for different time intervals.

Digital image of the Si wafer before and after Ag nanoparticle deposition (for 60 s) is shown in Figure 3a,b. A distinct and uniform color transformation can be observed in Figure 3b, which confirms uniform deposition of the Ag nanoparticles. The effect of Ag nanoparticle deposition on the optical properties of the Si was studied by measuring diffused reflectance spectrum in 300–1400 nm wavelength range. Figure 3c shows the comparative reflectance spectra on the unpolished side of a Si wafer, and Ag nanoparticles deposited on it for various time durations (30 s, 60 s, and 120 s). Due to the pre-textured surface, the unpolished side of Si showed a moderately low reflectance of 11% within 300–1400 nm wavelength in comparison to the reflectance of the polished side of the Si wafer ~48% in the same wavelength range. The reflectance shows a marginal increase in average reflectance with 30 s deposition of Ag nanoparticles. As observed from the SEM image shown in Figure 2a, Ag nanoparticle deposition performed for a time duration of 30 s resulted in less surface area coverage, which is also justified by the corresponding reflectance spectrum. In contrast to the reflectance spectrum of Si with Ag nanoparticles deposited for 30 s, the 60 s deposition shows a significant reduction in average reflectance to ~4% within 300–1400 nm spectral range. The significant reduction in the reflectance may be accounted for by the reduced interparticle (Ag–Ag) distance, which increases the occurrence of multiple internal reflections to ultimately trap the light inside. On further increasing the deposition time to 120 s, the reflectance was rather observed to increase again with an average value of 13.5% within the same wavelength range. The increasing trend is expected due to agglomeration of the Ag nanoparticles into dendrites, resulting in enhanced backscattering off the Si surface that eventually limits the occurrence of internal reflections and allows a major portion of incident light to reflect back [30]. Thus, based on the present optical reflectance results, chemical etching of the Si wafer using HF was considered to be optimum when Ag nanoparticles were deposited for 60 s. 

### 3.2. Structural Analysis and Optical Performance of Hierarchically Textured Si Substrate

**Figure 4:**
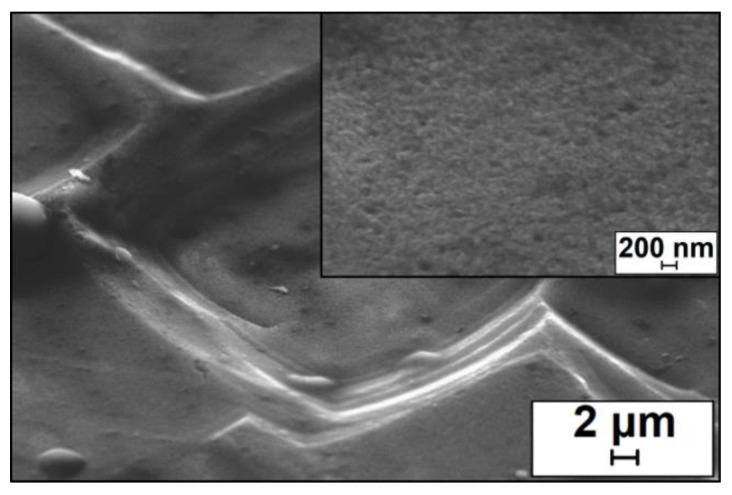
SEM image of the 5 s etched unpolished side of Si wafer after Ag nanoparticle removal (inset: magnified image showing the formation of nano-structured pits over the Si surface).

Figure 4 shows surface morphology of the hierarchically textured Si surface after Ag nanoparticle assisted etching in a mixture of HF and H_2_O_2_ for 5 s, followed by nanoparticle removal in HNO_3_. Etching in HF and H_2_O_2_ mixture was also performed for 10 s and 20 s that resulted in deeper nanopits and uneven erosion onto the unpolished side of the Si wafer (SEM images not shown). 

**Figure 5:**
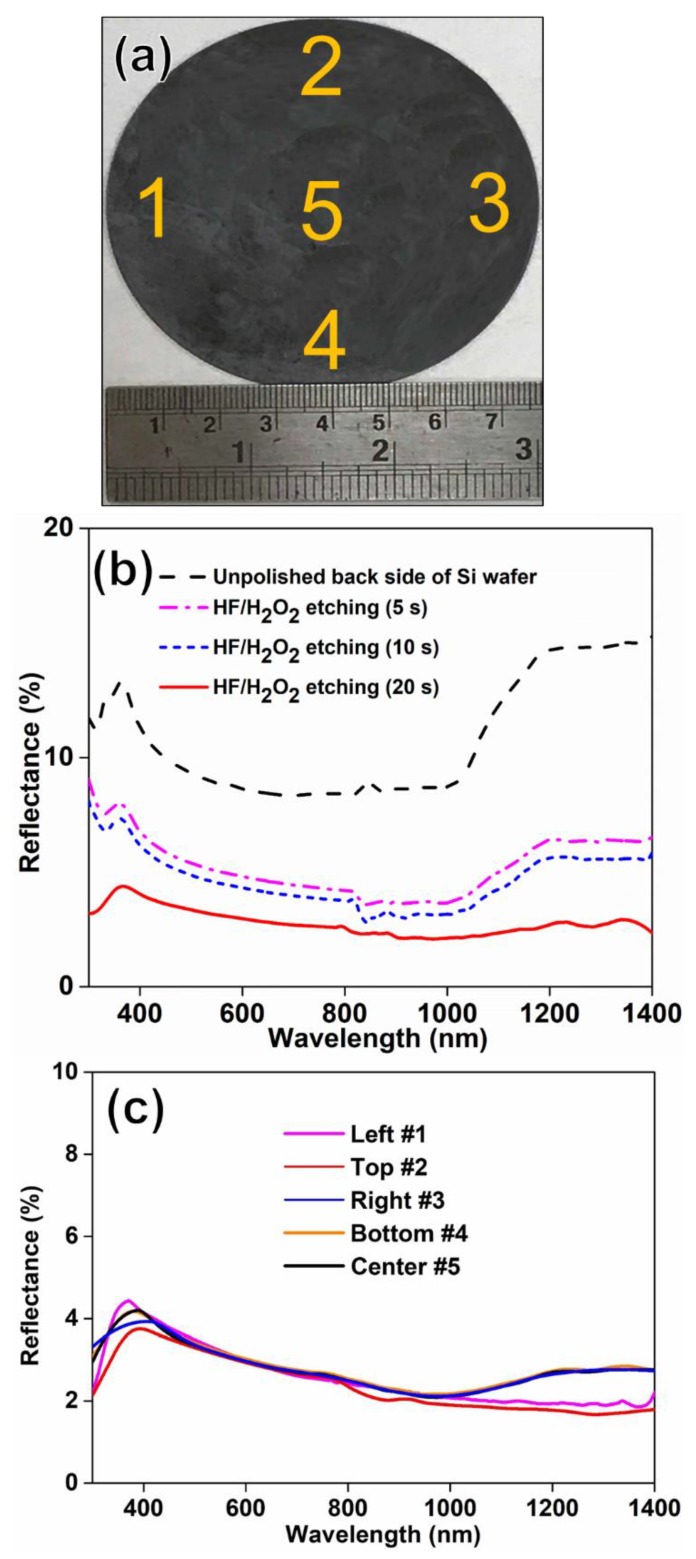
(**a**) Digital photograph of 5 s etched Si wafer (unpolished side) after removal of Ag nanoparticles. (**b**) Diffused reflectance spectrum of the unpolished side of Si wafer etched for different time intervals varying from 0 s to 20 s. (**c**) Reflectance spectra from five different regions of the 5 s etched Si wafer. Note: Reflectance spectrum shown is a mean of reflectance measured from five different regions of the wafer marked as #1 (left), #2 (top), #3 (right), #4 (bottom) and #5 (central area).

Digital image of the 5 s etched Si wafer (after removing Ag nanoparticles) is shown in Figure 5a. A distinct difference in the appearance of the Si wafer was observed in comparison to the wafer’s appearance as shown in Figure 3a,b, that was clicked before and after Ag nanoparticle deposition. Figure 5b shows the reflectance spectrum of the unpolished side of the Si wafer etched for 5 s, 10 s, and 20 s. Here, reflectance spectra were measured from five different regions of each wafer marked as #1, #2, #3, #4, and #5. The reflectance spectrum shown in Figure 5b is an average of five reflectance spectra as shown in Figure 5c. The mean reflectance for the 5 s etched Si was measured to be as low as ~2.7% within 300–1400 nm spectral range. Interestingly, as seen in Figure 5c, only a marginal variation of 2.7 ± 0.2 nm was observed in comparison with the reflectance observed from the different regions of the Si wafer. Further, as the etching time increased from 5 s to 10 s, and 20 s, an increasing trend in the reflectance was observed. The minor variation in the values of mean reflectance at different positions of the Si wafer indicates a good uniformity of the etched structures throughout the Si wafer surface. The reduced reflectance on the 5 s etched Si wafer surface may be attributed to the formation of nanotextures that tend to bend the light inwards and also trap the incoming light with multiple internal reflections, which lead to reduced surface reflection. Moreover, it is also understood from Fresnel’s theory that with variation in the volume ratio, the nano-structured pits of uniform depth act as a graded index layer between air and Si which effectively minimizes the overall light reflection [31,32]. However, optical results shown in Figure 5b indicate that a longer etching duration (>5 s) leads to a higher reflectance. This may have been generated due to uneven etch rates on the exposed (uncovered with Ag nanoparticles) and unexposed parts of the Si wafer as the H_2_O_2_ concentration used in our study was quite high (10 M). It is likely that the high concentration of H_2_O_2_ may have oxidized the exposed regions of Si as well, but at a much lower rate than the region underneath Ag nanoparticles catalyst, where oxidation and dissolution of Si occur preferentially.

Table 1 summarizes a list of literature on anti-reflection properties of textured Si surface. It is observed that the majority of the literature in Table 1 demonstrates reflectance varying from 1% to 5% in a textured Si surface, and the processing time is ranging from 1 h to 8 h [3,20,21,28]. In comparison to these reports, our process is significantly faster (only 65 s) to realize hierarchical texturing on Si that takes only 65 s. There are few pieces of literature that have demonstrated Si etching with a lesser time duration. For instance, Wang et al. [22] and Chen et al. [23] performed Cu metal assisted chemical etching on Si to improve the light trapping mechanism. Interestingly, this was a one-step process and the time duration for processing was ~15 min. A temperature higher than room temperature (50 °C) was required but the reflectance value obtained was 5% which is higher than our results (R ~ 2.7%). 

In addition, Yeo et al. [16] reported reflectance of etched Si as low as 1.6% in the spectral range of 300–1100 nm with an etch time of 13 min [16]. This process involves depositing a thin film of Ag ink on Si wafer, which was further sintered at a higher temperature (170 °C) for 3 min followed by metal assisted chemical etching for 10 min. In comparison to the method demonstrated by Yeo et al. our technique is still relatively rapid, and it is performed at room temperature only. Interestingly Yeo et al. demonstrated a reflectance of only 1.6% on the etched Si wafer, however, it is seen from their reflectance spectrum graph that there is a continuous increase in the reflectance value with an increase in the wavelength, especially beyond 1000 nm. Whereas, in our work, although the average reflectance value is slightly higher i.e., 2.7%; there is less variation in the reflectance with wavelength indicating a high broadband stability. 

Overall, it can be suggested that our proposed method is superior to the previous literature, as mentioned in Table 1, in terms of experimentation time, simplicity, and enhancement in anti-reflection property.

### 3.3. Structural and Optical Analysis of the Patterned PDMS Layer Using Hierarchical Si Template

**Figure 6:**
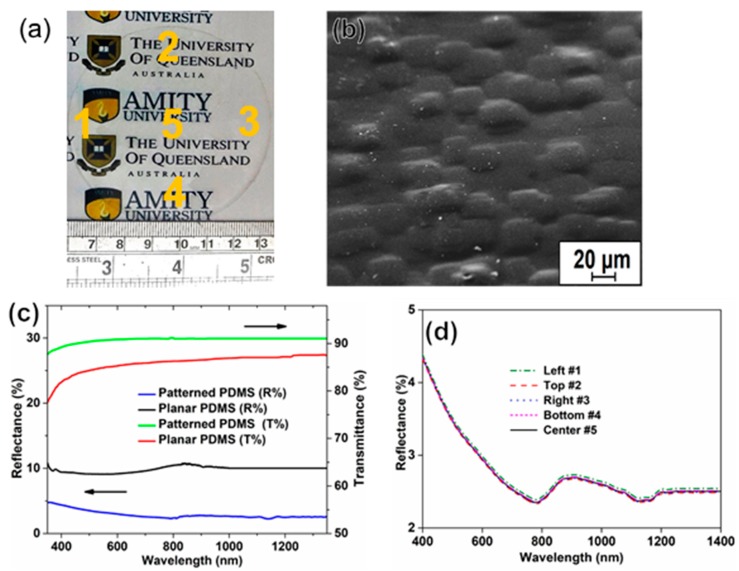
(**a**) Digital image of the patterned PDMS membrane (**b**) SEM image of the hierarchically patterned PDMS; (**c**) transmittance and reflectance spectrum comparison between planar and patterned PDMS. (**d**) Variation in surface reflectance across different regions of patterned PDMS; note: Point 1 is the extreme left area, point 2 is the top area, point 3 is the extreme right area, point 4 is the bottom area, and point 5 is the central area.

Solar cell modules are conventionally packaged with an encapsulation layer that protects it from environmental degradation and often improves the passage of incoming light by providing surface anti-reflection. Often polymer materials like ethylene vinyl acetate (EVA), thermoplastic polyurethane (TPU), and PDMS are employed as solar encapsulants [33,34,35]. PDMS has already shown its prospects for application as a solar encapsulant material [34] with inherent properties like high optical clarity, low UV degradability, low refractive index close to air (*n* = 1.41), hydrophobicity, etc. In this work, surface modification of a PDMS layer was performed using the as-prepared hierarchically textured Si wafer (shown in Figure 5a) as a template. The digital image of the patterned PDMS is shown in Figure 6a. The image clearly indicates the optical clarity of the PDMS layer. A comparative digital image of the patterned and plain PDMS is shown in Figure S1 (Supporting Information). The digital photographs clearly show the suppression of reflection on patterned PDMS relative to the plain PDMS, where clear areas of high reflection are observed and marked in yellow. The SEM image of the patterned PDMS layer is shown in Figure 6b. A negative imprint of the Si template is visible on the PDMS layer. Optical measurements were further performed on the patterned PDMS. A comparative optical spectrum (T% and R%) between textured and planar PDMS is shown in Figure 6c. Planar PDMS (without any texturing) shows an average transmittance of 85% in the wavelength range of 350–1400 nm, whereas the hierarchically textured PDMS displays an increased transmittance to approximately 90.7%. Increase in transmittance in the nano-textured PDMS layer is mainly due to the decrease in the reflectance, which is measured to be at 2.8% over a broad wavelength of 350–1400 nm (Figure 6c). Further, the surface homogeneity was evaluated by analyzing the surface reflectance across different regions of the patterned PDMS membrane (Figure 6d) in the same way as that was done for the etched Si wafer template. The mean reflectance value was measured ~2.8% as shown in Figure 6a which matched with the reflectance value acquired on the entire patterned PDMS membrane with only a minor variation of ±0.04 nm. This indicated that homogenous surface patterning occurred over the entire surface of the PDMS membrane. Overall, an improved optical property of the hierarchically textured PDMS indicates that its use as a solar encapsulant can bring distinct improvement in the efficiency and durability of an encapsulated solar cell. 

Table 2 compares the optical results on micro-nano-patterned PDMS reported in previous literature with our current approach. Dudem et al. (as mentioned in Table 2) demonstrated an average transmittance as high as 95% in the spectral range of 300–800 nm [36]. In this report, prototyping of PDMS was done from a 2 × 2 cm^2^ pyramidal micro-structured Si template that was prepared by etching for 40 min in an alkaline solution. It has been detected that wet anisotropic etching using alkaline solution often leads to the formation of hillocks when performed for a longer duration of time due to the evolution of a large amount of hydrogen bubbles in the entire process. Thus, the realization of a large-sized etched Si template using alkaline solution is not feasible due to lack of uniform surface texturing. Leem et al. performed LASER interface lithography and reactive ion etching (using SiCl_4_ plasma) to pattern nanoscaled features on Si that demonstrated optical clarity as high as 94.2% [37]. However, the gases used in the reactive ion etching process (e.g., SiCl_4_) are quite toxic and corrosive; and often are limited by re-deposition of non-volatile compounds, and needs specialized (expensive) equipment.

Other than the textured Si template, PDMS has also been patterned using templates like surface SU-8 epoxy resin [38], polymer [39], and glass [40]. PDMS patterned from a nanotextured SU-8 resin template offered optical clarity as high as up to 95% [38]. However, SU-8 resin is not very cost effective and has a low shelf-life. Senn et al. fabricated PDMS micro-lenses of 1 mm dimension by patterning from a nanotextured polystyrene template [39]. The resulted PDMS offered optical clarity ~90% over a broad wavelength range (350–850 nm). However, here the authors used a complex combination UV-nanoimprint lithography (UV-NIL) and thermoforming process to surface texture polystyrene. Using a surface modified glass template Galeotti et al. reported patterning of PDMS with an optical transmittance up to 94% in the wavelength range of 400–900 nm [40]. Here the surface patterning was performed on a 1.5 × 1.5 cm^2^ glass substrate by self-assembling polymer nanospheres of poly[9,9-di(2-(2-tetrahydropyranyloxy) hexyl) fluorene-alt-9,9-dioctyl fluorene] (PFOTHP). Although here the master template fabrication technique is facile as reported in reference [41], synthesis of nanospheres of PFOTHF is a complex process with an overall preparation time of four days. Moreover, self-assembly process on a large area often leads to the formation of micro defects. In our previous literature we used biomimicking process to pattern PDMS using rose petals. Biomimicking using rose petals demonstrated very high transmittance (~96%) and low reflectance (~3%). Although it is a green and cost-effective process, but it is limited by the size of the available template [42,43]. Biomimicking using rose petals can only pattern small size PDMS that may be suitable for encapsulating only small-sized PV devices (1 in. × 1 in.).

An overview of Table 2 shows that patterning of PDMS membranes has been widely explored on bio-templates as well as other templates like hard Si, textured glass, polyethylene terephthalate PET film, etc. Our current method of patterning PDMS on an easily obtainable hard Si template offers simplicity and enhanced optical properties on the patterned PDMS surface.

## 4. Conclusions

A rapid, simple, and low-cost fabrication process has been established for realizing hierarchically textured surface on the unpolished side of a Si wafer using an electroless Ag nanoparticle assisted catalytic etching. The surface texturing on Si can be performed on large areas with high uniformity. Further, the nanotexturing process demonstrated here using Ag nanoparticles assisted etching can also be replaced with nickel (Ni) nanoparticles as reported in the literature that can further reduce the cost of fabrication of hierarchical structured Si template. The hierarchical structuring on the unpolished side of the Si surface has demonstrated significant broadband anti-reflecting property that is strongly dependent on the dimension of the nano-structures. This anti-reflecting property of Si can be well utilized for several optical and optoelectronic device applications, such as photovoltaic devices, optical detectors, etc. Moreover, the simplicity of the fabrication process can initiate a high throughput production.

The hierarchically structured Si surface was further utilized as a hard template for prototyping thin PDMS membrane. PDMS replication using the hard Si template also supports multiple usages of the template till physically damaged, thus indicating high throughput patterning of PDMS. Excellent optical clarity and low broadband anti-reflecting features of the as-patterned PDMS makes it a potential candidate for use as an efficient solar encapsulant material. The anti-reflective property of textured Si also indicates the bright potential for applications as an anti-reflective surface for several optical and optoelectronic devices, such as photovoltaic devices and optical detectors.

## Figures and Tables

**Table 1:** Surface reflectance comparison on hierarchical structured and inverted micro-pyramidal structured Si.

Etching Technique	Appearance	Total Etch Time (s)	Bath Temperature (°C)	Average Reflectance (%)	Wavelength Range (nm)	Reference
Anisotropic wet chemical etching + Ag np assisted etching	Hierarchical structure (upright micro-pyramids with nanogrooves)	1 h 30 min	RT	4	450–2500	[3]
Ag np assisted etching	1D nano-structures	50 min	50°C	1.46	300–600	[14]
Ag ink assisted etching	Vertically standing disordered nano-structures	13 min	Ag sintering at 170 °C Etching at RT	1.6	300–1100	[16]
Wet chemical etching + Ag np assisted etching	Hierarchical structures (upright micro-pyramids with nano-grooves)	53 min	RT	3	300–900	[17]
Ni np assisted etching	Inverted micro-pyramid	1 h	RT	2.5	400–900	[20]
Cu np assisted etching	Inverted micro-pyramid	8 h	RT	1	300–1000	[21]
Cu np assisted etching	Inverted micro-pyramid	15 min	50°C	5	300–1000	[22,23]
Wet chemical etching + Ni np assisted etching	Hierarchical structure (upright micro-pyramids with nano-grooves)	73 min	RT	5	300–1100	[28]
Ag np assisted etching	Hierarchical structures	65 s	RT	2.7	300–1400	[*Current work*]

RT: Room temperature; np: Nanoparticle.

**Table 2:** Comparative summary of literature reports outlining the recent progress in the realization of patterned PDMS.

Surface Morphology of Patterned PDMS	Master Template Used and Fabrication Technique	Average Reflectance (%)	Average Transmittance (%)	Reference
Micro pyramidal patterned PDMS	Wet anisotropic etching using KOH on Si	4.7% (300–800 nm)	95% (300–800 nm)	[36]
Nanoscaled inverted moth-eye textured PDMS	Photolithography followed by RIE on Si	5.5% (350–800 nm)	94.2% (350–800 nm)	[37]
Nanopillar arrayed PDMS membrane	Pre-textured nanohole SU-8 template	-	94.5% (400–800 nm)	[38]
Nanoscaled moth eye featured PDMS stamp	UV-nanoimprint lithography followed by thermoforming process on polystyrene foil	-	90% (350–850 nm)	[39]
Randomly arranged nanoscale moth Eye patterned PDMS	Self-assembly of PFOTHP on glass substrate	-	94% (400–900 nm)	[40]
Hierarchical structure PDMS membrane	Biomimicking using rose petal	3% (400–1400 nm)	96% (400–1400 nm)	[42]
Hierarchical micro pyramid structured PDMS	Mechanical cutting to pattern inverted micro pyramid on PET film	5.2% (350–800 nm)	80% (350–800 nm)	[44]
Quantum dot filled nanotextured moth-eye patterned PDMS	Polystyrene beads assisted O_2_ plasma etching on Si	8–10% (300–800 nm)	-	[45]
Micro-structured PDMS membrane	Metal assisted wet chemical etching	2.8% (350–1400 nm)	90% (350–1400 nm)	*Present work*

## Supplementary Materials

The following are available online at http://www.mdpi.com/1996-1944/11/12/2586/s1.
