# Supplementary material for: Rapid Processing of Wafer-Scale Anti-Reflecting 3D Hierarchical Structures on Silicon and Its Templation
Source: Materials (Basel). 2018 Dec 18;11(12):2586. doi: 10.3390/ma11122586 (PMC6316734; doi:10.3390/ma11122586)
Supplement: Supplementary file 1 [file materials-11-02586-s001.pdf]

SUPPORTING INFORMATION

for

“Rapid Processing of Wafer-scale Anti-Reflecting 3D Hierarchical Structures on Silicon and its Templation”

Harsimran Singh Bindra [1,†], Jaikrishna [1,†] Tushar Kumeria [2,*] and Ranu Nayak [1,*]
[1] Amity Institute of Nanotechnology, Amity University Uttar Pradesh, Noida, UP, INDIA
[2] School of Pharmacy, The University of Queensland, Queensland 4102, Australia
[†] Authors have equal contribution
* Correspondence: rnayak@amity.edu; ranunayakbose@gmail.com
* Correspondence: t.kumeria@uq.edu.au

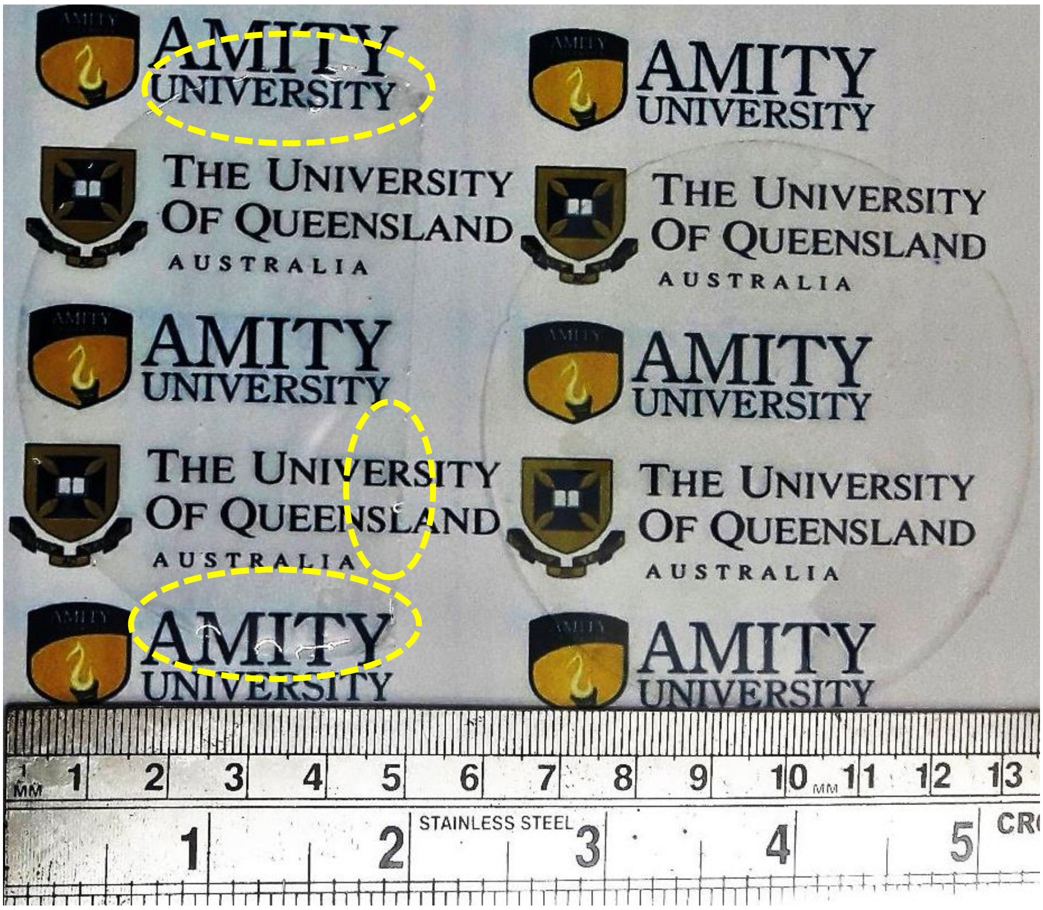

Figure S1: The Digital image comparisition of the plain PDMS (left) and the partterned PDMS (right).

© 2016 by the authors; licensee MDPI, Basel, Switzerland. This article is an open access article distributed under the terms and conditions of the Creative Commons by Attribution (CC-BY) license (http://creativecommons.org/licenses/by/4.0/).
